# “The missing piece in the puzzle” - Success factors and barriers for scale-up and sustainment of the Healthy School Start program

**DOI:** 10.1186/s13690-026-01835-0

**Published:** 2026-01-09

**Authors:** Jhon Álvarez Ahlgren, Kristi Sidney Annerstedt, Liselotte Schäfer Elinder, Susanne Andermo

**Affiliations:** 1 Department of Global Public Health, Karolinska Institutet, Stockholm, Sweden; 2 Centre of Epidemiology and Community Medicine, Region Stockholm, Stockholm, Sweden; 3 Department of Neurobiology, Care Sciences and Society, Karolinska Institutet, Stockholm, Sweden; 4 Department of Sport Science, Swedish School of Sport and Health Sciences, Stockholm, Sweden

**Keywords:** Implementation success, Scale-up, Sustainment, Health promotion, Parental support, Decision-makers, Children, Obesity, Overweight

## Abstract

**Background:**

Child obesity is a major global public health challenge. One way to reduce risk is through effective health promotion programs in schools that include parental involvement. However, programs often fail to be scaled up and sustained under real-world conditions. Therefore, it is necessary to study their implementation and study the perspective of decision-makers and school principals. The universal Healthy School Start (HSS) program, designed to promote healthy dietary and physical activity habits in children aged 5–7 years, was implemented in three municipalities in Sweden. This study aimed to identify and understand the success factors and barriers for scale-up and sustainment of the HSS program.

**Methods:**

This study used a qualitative explorative design. Individual semi-structured interviews were conducted with eight municipality leaders responsible for the school sector and eight school principals during 2023 and 2024. Data were analyzed using reflexive thematic analysis.

**Results:**

For a municipality to adopt and sustain the HSS program, dedicated leaders, in terms of health promotion, are crucial. Integrating the program into school routines and into the yearly quality assessment could support its sustainment. Barriers included challenges in prioritization of the HSS at the municipal level, perceived workload for school nurses, and staff and leadership turnover which could potentially reduce commitment to long-term program implementation. Facilitators and champions alleviated organizational challenges such as staff turnover. The feeling of support among staff was a key factor for successful implementation. To effectively promote health and prevent obesity, a multilevel and life-course approach involving several community actors was seen as necessary.

**Conclusion:**

Success factors for scale-up and sustainment included the appointment of dedicated leaders in the municipality serving as program facilitators by providing consistent support and follow-up during the first year, while barriers such as lack of program prioritization, high workload and staff turnover posed a challenge to the implementation. Program integration into the yearly quality assessment might be the missing piece of the puzzle needed to achieve sustained implementation at scale. These findings are likely applicable in settings with a decentralized school system similar to Sweden’s.

**Supplementary Information:**

The online version contains supplementary material available at 10.1186/s13690-026-01835-0.

**Table:** 

Text box 1. Contributions to the literature
• This study adds evidence on the importance of sustained leadership support and systematic follow-up in maintaining staff motivation, commitment and perceived capability to deliver a health promotion program, key factors for successful implementation at scale.
• New knowledge regarding how shifts in municipality priorities, leadership turnover, and lack of leadership support can affect program continuity, even when initial implementation was successful.
• The findings suggest that program integration into existing routines and mandated inclusion of the program in the yearly quality assessment work of the school could strengthen implementation and promote long-term sustainment.

## Background

The rising global prevalence of overweight and obesity among children and adolescents is a major public health concern worldwide that has a large impact on the burden of disease [1, 2]. The main causes of the increase in overweight and obesity over recent decades have been changes in the food and physical activity environment, leading to an imbalance in energy intake and expenditure [3, 4]. In Sweden, the prevalence of childhood overweight and obesity is also increasing, and in 2022, approximately 23% of children aged 6–9 years had overweight (15,6%) or obesity (7,2%) [5]. The health risks associated with a high body mass index (BMI) are well documented and are likely to increase with age if the condition is not reversed before puberty. These include a greater risk and earlier onset of type 2 diabetes, cardiovascular diseases and several forms of cancer [4, 6]. Similarly, these issues impose a substantial economic burden and reduced productivity in society [7, 8].

Health promotion programs addressing both diet and physical activity habits can reduce the risk of overweight and obesity in children [9, 10]. Importantly, these programs are most effective when initiated at an early age [9, 11], delivered at school [9, 12, 13] and actively involve parents [9, 14, 15]. However, effective interventions often fail to be scaled up and sustained over time [16, 17]. This is unlikely to be due to a lack of intervention effectiveness [18], but rather due to the complexity of the process of transitioning interventions from research to practice in real-world conditions [17], insufficient focus on implementation outcomes and/or a lack of understanding of the context by program evaluators [16, 19, 20].

The Healthy School Start (HSS) program, based on Social Cognitive Theory [21], was designed to promote healthier lifestyles in young children by engaging both the school and parents or caregivers and their children during one school year. This program is delivered by teachers and school nurses in compulsory pre-school class or first grade (children aged 5–7 years). It is universal, meaning that all children receive the program. The HSS program includes four components [21]: (1) A health information brochure to parents; (2) motivational interviewing (MI) health talks with parents performed by the school nurse; (3) structured activities for children within the classroom performed by teachers with home assignments to be completed by children and parents; and (4) a type 2 diabetes (T2D) risk self-test for parents with a recommendation to seek medical advice in primary health care (PHC) if needed. The goal of the HSS is to create a supportive environment that helps families develop and maintain healthy habits and prevent obesity. The program has been evaluated in three randomized controlled trials and has shown positive effects on diet and physical activity [22–24]. Moreover, based on a pooled sample from all three RCT trials, there was a significant reduction in BMI z-score in children with obesity compared with controls after the one-year intervention [25].

Based on these positive findings, the research team developed the IMPROVE implementation study to scale up the HSS from individual schools to entire municipalities over two school years. In Sweden, public schools are regulated by national school law (§ 2020 − 800) and the national curriculum (Lgr22), but administered at the municipality level across 290 municipalities [26]. Previous process evaluations of the HSS program have focused on the experiences of parents [27], children [28], and school staff involved in program delivery [29]. However, little attention has been given to stakeholders in leadership positions, despite their critical role in program adoption and sustainment.

Understanding the leaders’ motivation and capacity to implement the program is essential for successful scale-up. According to Weiner’s Theory of Organizational Readiness for Change (TORC) [30], readiness reflects the collective commitment and confidence of organizational members to integrate a new program into routine practice. Organizational readiness has been associated with positive implementation outcomes [31–33]. In schools, leadership support and staff engagement are particularly influential for program fidelity and long-term sustainment. Including the perspectives of municipality leaders and school principals can also demonstrate important outer-context factors, such as policy, funding and local politics [34, 35] that strongly affect implementation and have been linked to sustainment [36], yet remain understudied [37]. This oversight may be due to the lack of research studies including decision-makers involved in the adoption and implementation of health promotion programs [37]. This study was conducted after the municipality wide implementation of the HSS where decision-makers were central in both adopting the program and facilitating collaboration among school staff [38].

Guided by the research question “What are the critical determinants for scaling up and sustaining the HSS program”, the aim of this study was to identify and understand success factors and barriers for scale-up and sustainment of the HSS program from the perspective of decision-makers (municipality leaders, school principals and vice principals).

## Methods

This study used a qualitative explorative design. Data were analyzed using reflexive thematic analysis within a constructivist paradigm [39], with the aim of capturing how stakeholders in leadership positions perceived program implementation [40]. This methodology allowed for an in-depth exploration and understanding of the participants’ perspectives, and subsequent identification of success factors and barriers, acknowledging how researchers participate in shaping knowledge in their interaction with participants and data [41]. Furthermore, the Theory of Organizational Readiness for Change [30] was applied. This approach provided insights that can assist practitioners and implementation scientists in operationalizing and applying our findings in similar settings. This study followed the Consolidated Criteria for Reporting Qualitative Studies Questionnaire (COREQ) (see Additional file 1) [42].

### The IMPROVE study

IMPROVE is a hybrid type III study with a 2-arm parallel cluster randomized design. It compared the effects of two bundles of implementation strategies (Basic and Enhanced bundle) on the fidelity to the HSS program components [43]. Strategies in Bundle 1 (Basic) included conducting local consensus discussions, distributing educational materials, organizing school health teams, peer-assisted learning, changes on the school environment, and preparing families to be active participants in the program. Bundle 2 (Enhanced) added external support in the form of education in health promotion for staff by the research team, network weaving between the school and the PHC, coaching provided by the research team including feedback from parents and ongoing follow-up in addition to the Basic strategies. The IMPROVE study took place in three municipalities in Stockholm County, Sweden, with a higher prevalence of childhood obesity than the county average [43].

### Setting

The IMPROVE study was conducted in 45 schools from three municipalities (hereafter M1, M2 and M3) in Stockholm County. Two of the municipalities (M1 and M2) have mixed socioeconomic characteristics, while one (M3) is socioeconomically disadvantaged, representing real-world variation in the school system capacity and community needs [43]. The municipalities’ characteristics are shown in Table 1. M1 and M2 started implementation in 2021, whereas M3 decided to start in 2022 because of the COVID-19 pandemic.

**Table 1:** Characteristics of the municipalities

	Municipality 1	Municipality 2	Municipality 3	Stockholm County
Population (August 2024) [44]	113 951	50 273	101 209	2 467 195
% unemployed (September 2024) [45]	7,2%	8,6%	11,1%	6,9%
% of 16–74 year olds with higher education [46]	26%	18%	19%	31%
Overweight among 4-year-olds born in 2017 [47]	9,4%	11,7%	11,4%	9,1%
Number of participants included in the study	5	5	6	16
Number of schools in the IMPROVE study	13	13	19	-

### Participants

Municipality leaders in managerial or supervisory roles within the fields of child health, education or both at the central administration and who had participated in the IMPROVE study were purposefully selected for this study [48]. All the school principals were also invited for interviews. Participant recruitment took place between 2023 and 2024, when M1 and M2 had completed the study while M3 was still running it. School principals in M3 were invited during visits to schools for data collection as part of the IMPROVE study. Participants from M1 and M2 were contacted via email, two telephone calls and a final email invitation.

Ten managers in the municipalities that had been involved in the HSS program were invited; eight of them were interviewed, while two declined because of a lack of time. In the included schools, 43 principals and 31 vice principals were invited. Most schools had both a principal and a vice principal, but usually, only one of them was involved in the program. A total of eight principals or vice principals (hereafter referred to as principals) still working at the schools were interviewed. The sample size for this study was determined by applying the concept of information power proposed by Malterud et al. [49]. Information power provides a nuanced and context-sensitive approach to sample size determination in qualitative research, emphasizing that the more relevant information participants provide in relation to the study aim, the fewer participants are needed.

Accordingly, the aim of this study is narrow, focusing specifically on the scale-up and sustainment of one health promotion program, the HSS program. This increases information power by allowing for a focused exploration of implementation dynamics within a defined context. The sample specificity was dense, as participants were purposefully selected based on their direct involvement in the decision-making and implementation of the program in their respective municipalities and schools. This targeted recruitment ensured that collected data was relevant to the study aim, in contrast to a broader sample that might have included all school principals or municipal leaders with general responsibilities in health or education.

The study is informed by the TORC, which provided a conceptual ground for discussing the data. The use of an established theory enhances information power, supporting a deeper analysis and guiding the identification of relevant themes. The quality of dialogue during the interviews was high. All participants were highly educated professionals working in leadership positions, which contributed to articulate, reflective, and fluent conversations. No power imbalances were perceived, and the interviews were conducted in settings chosen by the participants, further supporting open and rich dialogue.

The analysis followed a reflexive thematic approach within a constructivist paradigm, allowing for nuanced interpretation of the data. The iterative coding and theme generation process, combined with collaborative reflection among researchers, contributed to analytical depth and rigor. Based on the narrow aim, dense sample specificity, use of established theory, high-quality dialogue, and robust analysis strategy, the sample of 16 participants was sufficient to achieve high information power. All participants were female, aged between 36 and 64 years, held university degrees and had been working in their current position for five years on average.

### Data collection

An interview guide (see Additional files 2 and 3) was developed to explore critical factors for implementation, scale-up, and sustainment based on the Intervention Scalability Assessment Tool (ISAT) [50]. The ISAT is designed to help policy-makers and practitioners to systematically evaluate the adequacy of health interventions for large scale implementation. This tool was chosen because it was developed for use in high-income countries, such as Sweden, provides a structured and adaptable evaluation, and directly aligns with the study’s focus on implementation, scale-up, and sustainment. The interview guide was piloted among the research team and other colleagues, resulting in adjustments. After the first interview with a municipality leader, the guide was slightly modified by removing two redundant questions. This interview was included in the study.

The recruitment and interviews were conducted from December 2023 to July 2024. All the interviews were held in Swedish by the first author (JAA). The co-authors were present in some of the interviews: KSA in three interviews, LSE in three interviews and SA in two interviews. The research team brought diverse professional backgrounds to the study. LSE has extensive experience in complex interventions and implementation research in the school setting, KSA has extensive experience in the implementation of complex interventions across a wide range of contexts and possesses strong expertise in qualitative research methods. Both had prior contact with municipality leaders through their involvement in the IMPROVE study. JAA joined the team in June 2023 and had no prior involvement with the municipalities. He took the lead role in communications and conducted all interviews, thereby assuming an outsider position that contributed to neutrality and openness in the data collection process. SA, with a background in social anthropology, and expertise in qualitative methods and school-based interventions, provided additional reflexivity and depth to the analysis. All authors have a public health background and have worked in different socioeconomic and cultural contexts.

Two out of eight interviews with municipality leaders were conducted online, whereas the other six were conducted at the participants’ offices. Three out of eight interviews with school principals were conducted online, upon the participants’ request, and the other five at the schools.

### Data analysis

The interviews were audio recorded and transcribed verbatim using Microsoft Word and were manually revised and corrected. The data were analyzed with an inductive approach using reflexive thematic analysis according to Braun and Clark [40, 51]. First, JAA listened to the interview recordings and read through the transcripts and the field notes to become familiar with the data. Second, one interview was coded inductively by JAA and SA together and discussed with KSA and LSE to establish consistency. Thereafter, the rest of the interview data were coded line by line by JAA following an iterative process in which the progress was discussed with all the authors and the interview data were revisited when needed. After the codes were defined and condensed explanations giving meaning to the codes were written, these were grouped based on their semantic patterns describing the program implementation process, consistent with the field notes and subsequently preliminary sub-themes were generated. These were reviewed by JAA and discussed with the research team for consistency with the codes and their meaning. Summaries and descriptions of the preliminary sub-themes were written, followed by the selection of a quote from the interview transcripts to explain the meaning of each sub-theme. The sub-themes were subsequently transferred to a spreadsheet and charted into a framework matrix containing: (1) Sub-themes; (2) codes and (3) condensed descriptions of the codes organized by participant. This facilitated data visualization and the reflexive process during the analysis. Afterwards, the final themes were generated by interpreting the codes and repeatedly returning to the interview transcripts to ensure that the sub-themes captured what was expressed by the participants. Finally, related sub-themes were further synthesized based on their descriptions through collaborative and iterative reflection among the authors and grouped to generate multi-layered themes containing the most relevant meanings from the data. The themes were provisionally labeled, and then the authors discussed and reflected on the themes’ name and meaning to refine them and reach a consensus on a definitive version.

Each municipality included schools assigned to both the Basic and Enhanced implementation bundles of the IMPROVE study. Municipality leaders were not involved in bundle-specific strategies and instead oversaw all schools as part of their routinary duties. Since the unit of analysis in this study was the individual municipality leader or school principal, and no interview questions explicitly addressed specific experiences to the IMPROVE implementation bundles, a comparative analysis between bundles was not performed.

### Ethical considerations

Participants were informed about the aim and rationale of the study via email before the interview, they were given a few minutes to read the information sheet again. The participants provided informed consent to participate in the study prior to the start of the interview in writing or verbally for those who were interviewed online. Approval for this study was provided by the Swedish Ethical Review Authority #2021–02267 and #2023-05658-02.

## Results

**Fig. 1:**
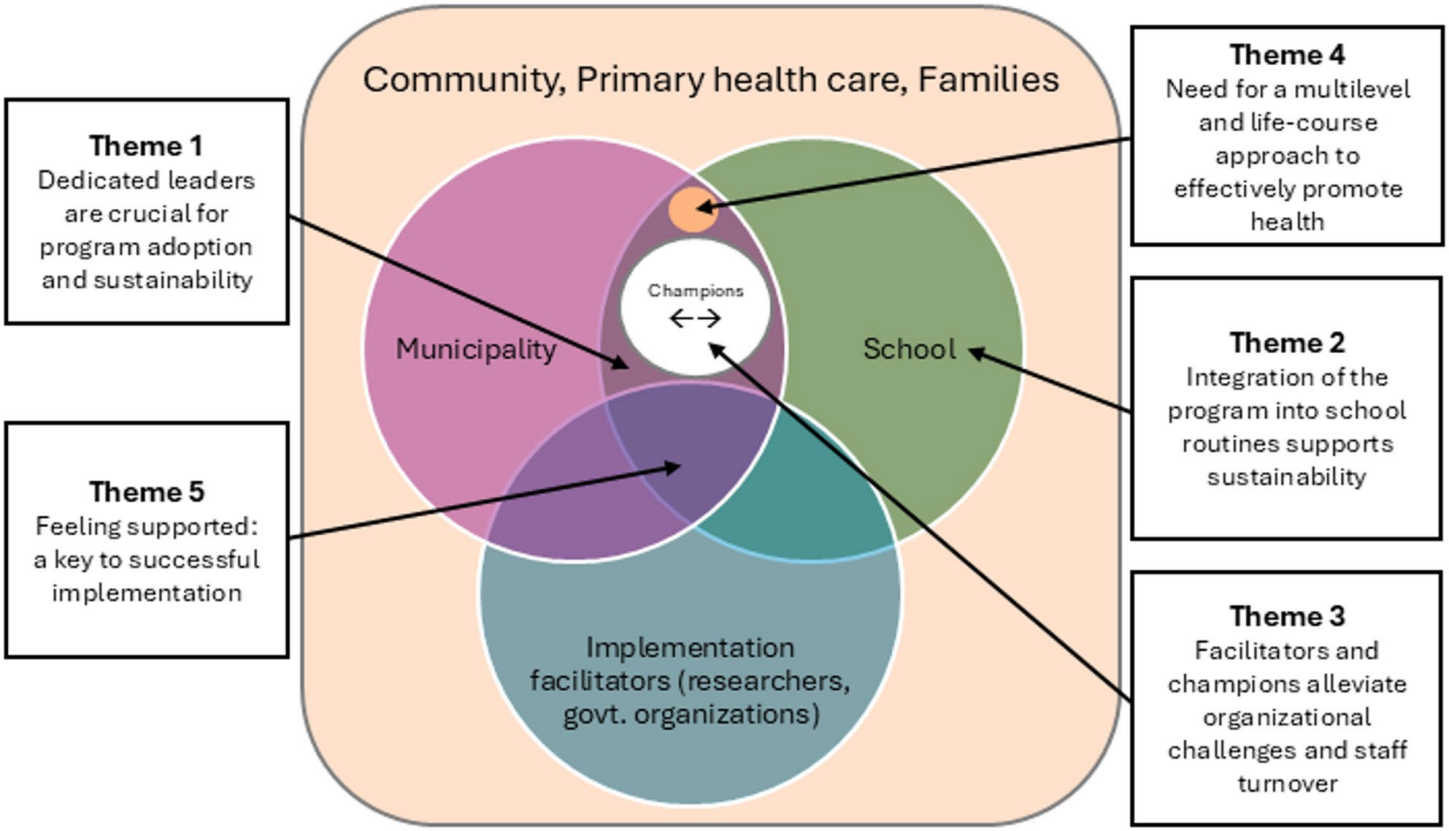
Themes and interactions in the context of stakeholders and the community influencing program implementation

Five themes were generated from the interview data and contextualized, as shown in Figure 1. The themes are related to both the individuals involved in program implementation and community factors. Figure 1 illustrates the interaction between the respective actors at various levels (i.e., school, municipality, and implementation facilitators) and how the themes relate to or describe those interactions. Implementation facilitators were those who delivered implementation strategies and support to schools and municipalities.

### Theme 1. Dedicated leaders are crucial for program adoption and sustainability

The participants noted that the municipality’s agenda and priorities were largely shaped by its leaders and politicians. Having municipality leaders who were dedicated to health promotion and prevention and who were engaged in the implementation was critical for program commitment and sustainability beyond the first year. Dedicated leaders who were committed to implementing the HSS program made efforts to monitor the work through the school principals and nurses, and offered support to the schools. While evidence demonstrating the intervention’s effectiveness was considered important, it did not appear to be the primary factor influencing a municipality’s decision to implement the HSS program. Therefore, identifying key actors who were dedicated to health promotion greatly enhanced the decision-making process regarding program adoption (Fig. 1).

#### Sub-theme 1.1: Complexity and opportunity in reaching buy-in consensus

The decision-making process for implementing the HSS program varied among participants. Some described it as a democratic process that involved school principals aimed at reaching consensus:

***“****We presented the HSS program to the principals… Then*,* we discussed the question*,* and in the next meeting*,* we had a decision to make*,* so it was a democratic process”* Municipality leader K08

In contrast, others mentioned that decisions occurred solely at the municipality level, with principals informing only after the decision to adopt the program had been made. When principals were included in the decision-making process, there could be reluctance to adopt the program; however, this involvement also created a sense of ownership, which increased their motivation. The process was described as complex and driven by people with an interest in health promotion, who encouraged adoption.

#### Sub-theme 1.2: Balancing municipality priorities and program implementation

While most participants considered that the HSS program aligned with the municipalities’ policy, the curriculum and school agenda, it was deemed necessary for widespread adoption to explicitly integrate the program into the municipality’s goals and describe a follow-up mechanism. The participants said that the municipality’s priorities were determined by the policy of the ruling political party, and this may or may not have allowed municipality leaders to prioritize the HSS program. For example, in M1, some schools discontinued the program after implementation support by researchers had ended. Participants cited reorganization in the municipal structure, budget and staff cuts which reduced their capacity, as well as pressure to prioritize crime prevention among youth, as reasons that prevented them from keeping the implementation at the top of their agenda.

Other principals thought that it was possible to include the program in municipal policy:

*“We need to take into consideration our political goals*,* I think. And I can say that in the past years we have had many goals to fulfil. Then it (HSS) needs to be one goal that fits into the work that we are doing so we can see that it is the missing piece of the puzzle”* Principal R22

### Theme 2. Integration of the program into school routines supports sustainability

The participants reported that school staff, especially school nurses, generally have high workloads. Municipality leaders emphasized that health talks with parents using MI were a valuable and impactful component that was not overly burdensome. However, other participants at the municipal level noted that it was burdensome for nurses, particularly when an interpreter was needed for non-Swedish speaking parents.

Program integration into school routines should involve the whole school, as highlighted by a principal. They suggested adding a specific question about the HSS program in the yearly quality assessment questionnaire administered to all schools throughout the municipality. This would contribute to establishing clear parameters of success in terms of the work with the HSS program, serve as a follow-up mechanism and thereby contribute to more effective implementation and sustainment beyond the first year.

#### Sub-theme 2.1: Program integration and collaboration among staff can defuse school-based challenges

The participants thought that integrating the HSS program into the school’s routines was crucial to ensure continuity and responsibility for the program as expressed below:

*“I think this (HSS) would need to be a part of our core activity program so it can feel like we have time for it. If that is case*,* then you see it in a different way”* Municipality leader K01

The program activities were reported to be easier for teachers than for nurses because of the nature of their activities. Teachers could easily integrate HSS topics into the existing curriculum with subjects related to the human body. While nurses had a health talk with every family, they did not necessarily use the MI technique at all health visits:

*“The teachers could share the work because they were three in each division that worked with one group of students. The work was more evenly distributed. However*,* of course*,* since the school nurse had to work individually*,* that meant a larger workload”* Principal R23

The HSS program promoted collaboration among school staff, as emphasized by a principal. However, they felt that the program should also involve other school personnel, such as those working in the school cafeteria and after-school care. Implementation could have been influenced by school size, staff situation, and socioeconomic factors in the area. A municipality leader suggested that in smaller schools, the contact between staff is tighter, and thereby, the program could work better; however, allowing schools to adapt the program to their situation and needs would help address these differences.

#### Sub-theme 2.2: Lack of consensus on whether health promotion is part of the school staff’s duties

Many participants acknowledged that school staff faced significant pressure and time constraints, making it difficult to add new tasks to their already extensive list of responsibilities. A principal thought that discussing eating habits fell outside the teachers’ duties: *“To talk about*,* for example*,* about diet and eating disorders is not something we usually do. It is outside of our duty”* Principal R23.

Some municipality leaders noted that the use of the MI technique in health talks with parents was challenging to perform with non-Swedish born parents: *“It is difficult to conduct motivational interviews and especially using this method when you need to have an interpreter.”* Municipality leader K04.

Municipality leaders described MI as important and as having the potential to have a positive impact on families: *“I think it is the school nurse with her (motivational) interviews with caregivers who can make a difference.”* Municipality leader K01.

### Theme 3. Facilitators and champions alleviate organizational challenges and staff turnover

Significant barriers to implementation included a lack of staff and high turnover at both the municipality and school level. Conversely, school nurses made the difference between discontinuing or continuing the program after the end of the study. Principals described them as dedicated to the program, as some nurses assumed an informal leadership role. Similarly, in one municipality, leaders facilitated knowledge exchange by providing a digital platform for staff to share material and support each other.

#### Sub-theme 3.1: Leadership engagement boosted staff motivation

When leaders at the municipality and school level prioritized the program in their routine meetings, it facilitated implementation. For example, one municipality, in the first year of the HSS program, provided extra support to school nurses in their regular monthly meetings where they practiced problem solving and role playing. Furthermore, similar to facilitators in the municipality, informal champions in schools also contributed to staff motivation and support (as shown in Fig. 1).

*“I know that many schools got the possibility to decline working with the HSS program… However*,* we have a school nurse that is passionate about the program… She felt like despite all the difficulties and challenges she still wanted to deliver the program and decided to take a central role in the implementation and to be the contact and support for our teachers”* Principal R22

Successful implementation depended on the motivation of the municipality leaders and school staff, as well as their belief in the program’s benefits. At times, this made the implementation process dependent on motivated municipality leaders or school staff who took the initiative to support and encourage their colleagues. For example, informal champions used their influence to encourage adoption by convincing principals to continue the program in schools once the study had ended.

A lack of engagement from school leadership could cause stress among staff and thereby hinder implementation. In contrast, an important factor contributing to sustainment was that the principal or adjunct principal took the lead responsibility for the HSS program at the school.

*“We were very clear that it was the principal or vice-principal that must hold the responsibility for the HSS program… It is easy that it falls between the cracks if it’s a teacher who has the responsibility. Therefore*,* I think the principal is very important and a key person in this.* Municipality leader K08

#### Sub-theme 3.2: Staff shortages, turnover and the enabling role of informal champion

Common challenges included a lack of staff, turnover and organizational instability at both the school and municipality level, which in turn hindered sustainment of the program. Changes in the municipality organization and leadership positions were perceived as stressful, which could have generated reluctance among school leaders and staff to engage further with the program.

*“It became voluntary (to work with the HSS program)*,* so several principals said no right away. I don’t know the reason. Partly perhaps because they did not have a permanent school nurse or the nurse had been replaced.”* Municipality leader K05

However, dedicated staff (informal champions) motivated colleagues, followed up on their work, thus contributing to alleviating the stress generated by the described challenges.

### Theme 4. Need for a multilevel and life-course approach to effectively promote health

A strong success factor for a municipality to adopt the HSS was the program’s universal approach for health promotion and obesity prevention (all children in the school and their caregivers receive the same program).While parental engagement was considered an important core principle of the HSS program, a multi-faceted approach ‘working on different levels’ and involving external actors was deemed key for success in promoting healthier habits in children and families. The HSS program alone was not perceived as sufficient in itself to prevent obesity and overweight in society. Instead, a combination of societal actions and community collaboration, emphasized through the engagement of additional community actors such, e.g. sports clubs, was considered necessary.

#### Sub-theme 4.1: Both caregivers and school staff are essential for better child health

Participants emphasized the critical role of parental involvement in fostering positive health outcomes among their children. They rationalized this by explaining that parents are primarily responsible for facilitating opportunities for physical activity and providing healthy and nutritious food choices at home: *“It is really a family problem*,* it is not really the child*,* you need to improve the behavior of the whole family”.* Municipality leader K01.

Participants expressed that health promotion and prevention should start even earlier, with children under five, and continue all the way to high school. The universal nature of the HSS program was understood as important for mitigating socioeconomic inequalities in health. Parental support, although sometimes challenging due to language barriers and a lack of engagement among caregivers, was considered important for promoting healthy habits effectively, and school staff played an important role in engaging parents according to a principal:

*“You can motivate the children all you want*,* but it is mom and dad who buy the stuff (food). It is mom and dad who pay for extracurricular activities or exercise and so on. Therefore*,* it is them (caregivers) who have the power to decide.”* Municipality leader K02

#### Sub-theme 4.2: Multisectoral action as a complement to the HSS program

Participants emphasized the need for collaboration with external actors, including primary health care, civil society, sports clubs, and food retailers, to promote healthy habits in the community, noting that schools could not achieve this alone.

*“Community engagement is needed when it comes to caregivers. Then*,* you also have grocery stores*,* sports associations… and even the cultural scene… We want to develop activities for children”* Municipality leader K04

Societal engagement, links to primary healthcare and civil society and the application of a life-course approach were also considered essential for promoting healthy habits equitably. However, participants thought that establishing such collaborations might require effort and external support.

### Theme 5. Feeling supported: a key to successful implementation

Feeling supported throughout the implementation process involved several elements across different phases. The ongoing consultation and training sessions offered by the research team to schools in the Enhanced bundle as part of the IMPROVE study eased the initial efforts by the school staff to initiate the program and increased the feeling of support. To succeed with the program implementation, the participants expressed the need to plan the work in advance as this resulted in reduced stress among the staff and increased understanding of the program. A central part of this process was municipal support with logistics (e.g., printing and sending out materials). The presence of researchers at the school to provide credibility was also believed to be important for improving perceived program effectiveness and staff engagement.

#### Sub-theme 5.1: Time for planning and preparation mitigates initial implementation barriers

There was an initial perceived learning curve and considerable effort needed to implement the HSS program; however, the staff became more comfortable with the process after the first year. Leaders from the different municipalities had diverse experiences regarding the initial efforts, which were driven by specific circumstances in their context, such as ongoing organizational changes. The ability to plan program implementation in advance and integrate HSS tasks into routines was crucial to avoid stress and reluctance among already overloaded school staff. Looking back, receiving program information one year in advance and training the staff six months before the start of the academic year, as well as clearly assigning roles, were seen as positive steps toward ensuring sustainment of the program after the first year.

*“This year*,* it (HSS) has worked pretty well*,* a little better than the first year*,* but it also depends a little on how the teachers work with the program.”* Principal R26

#### Sub-theme 5.2: Different strategies increased feelings of support among staff

The program implementation in the three municipalities involved implementation strategies provided by the research team both before and during the start of the academic year. In M3, municipality leaders also provided extra MI support for school nurses and created an online platform to share materials and updates. The participants agreed that these activities were positively received by school staff, facilitated collaboration between schools, improved the sense of support, and contributed to program sustainment. A principal described the extra support received from the municipality:

*“The municipality collects the data in the beginning of the academic year such as which teachers are in 1st grade next year? How much material do they need? … Then*,* the municipality sends out information about the upcoming meeting at the beginning of the academic year… I think that is very important that there is someone at the municipality that holds the work together*,* so no one misses anything”Principal R25*

Participants posited that successful implementation of the program involved bringing up the importance of all intervention components together and having local coordinators or facilitators oversee logistics, regular follow-ups, consultations and staff training. Consequently, school principals thought that the presence of researchers at the school could boost staff motivation and engagement, with lectures for staff and parents facilitating implementation.

## Discussion

The aim of this study was to identify and understand the critical success factors and barriers for the scale-up and sustainment of the HSS program at the municipality level. Our findings show that for a municipality to adopt the HSS program in all or most schools, it is important to identify and appoint relevant municipality leaders responsible for health, education or both as facilitators who are dedicated to health promotion and prevention. Moreover, viewing the program as part of the curriculum, may increase consistency in following it up, which in turn could enhance the staff’s change commitment, by aligning the program with their priorities and improving motivation to sustain its implementation. Incorporating the HSS program in the yearly quality assessment questionnaire could represent a crucial and necessary step for ensuring its sustainability. At the school level, consistent follow-up and coaching, especially during the first year, as well as having established criteria for success and facilitating program integration into existing routines improved implementation and sustainment. These factors, from a theoretical point of view, would also support change efficacy, as providing support early in the implementation process can help build confidence in the staff’s perceived capability to deliver the program. Municipality leaders, particularly in M3, played an important role as facilitators, enabling schools to effectively implement the program. They supported the creation of a platform for knowledge exchange among staff, followed up on the program work and created spaces for the staff to develop problem-solving skills related to HSS activities.

Our findings highlight the impact of engaging with dedicated leaders on both the implementation and long-term sustainability of the program. In one municipality (M3), leaders who made the HSS program mandatory for all schools successfully encouraged school staff, including principals, to promote healthy habits and emphasized the importance of preventing obesity and overweight. These findings are similar to those of previous research in school settings, underscoring the importance of support and commitment from high-level leadership [52, 53]. Leaders who have not only the desire but also the confidence to work effectively with the HSS program in their municipality or their school express a high level of Organizational Readiness for Change. Therefore, identifying and engaging these leaders can be key to successful implementation [30]. Conversely, if leadership commitment is lacking, it may be advisable to delay program initiation and focus on strengthening readiness for change.

In the HSS program, school nurses allocate an additional 20–30 min per family for health talks using MI [43]. We found mixed opinions among municipality leaders regarding the burden of MI, some consider it time-consuming, while others do not. Despite these differing views, evidence suggests that MI can serve multiple purposes in the school context, particularly in meetings with older students. It also enhances school nurses’ sense of gratitude and job satisfaction when promoting health [54]. However, time constraints and difficulties in fitting health promotion activities into staff duties [55] as well as challenges to perform MI [56] have been identified as barriers in school-based studies.

Integrating MI, as part of the HSS, into the school nurses’ core activities, could be advantageous, provided sufficient time and resources are available [54]. This integration may also contribute to the sustainability of the program. Given the many mandatory duties of school nurses, e.g., administering vaccinations, embedding MI within existing activities may as well ensure long-term implementation [54]. These findings are consistent with earlier research showing that integration into school routines facilitates HSS implementation [53]. A recent review further supports this, showing that the impact and sustainment of school health interventions depends on the integration of individual-level components into school-level routines and the inclusion of families [57]. Likewise, consensus regarding the teachers’ role in health promotion among principals and teachers can help clarify responsibilities and facilitate the integration of the HSS, particularly the classroom component. This could reduce barriers to implementation and facilitate leadership directives from the municipality to implement the program.

In the context of the TORC, the potential integration of the HSS into the yearly quality assessment questionnaire [58] could also help clarify staff responsibilities. In turn, this clarity could influence the staff’s perceived capability to perform these tasks, thereby enhancing their motivation to engage in the program [30]. Similarly, aligned with the theory, this integration can represent a move toward institutionalization of the practice (i.e. HSS program implementation) and support sustainment. A study on principals’ perceptions of health-promotion programs in Norwegian schools indicated that adding the program to the curriculum and recognizing its positive effects can lead to commitment to sustain the practice and thus make the program less vulnerable to staff turnover [59].

Facilitation has been described as a widely used strategy leading to successful implementation [60, 61]. Implementation facilitators are professionals who help organizations implement changes and contribute to tackling challenges, offering problem-solving and enhancing adoption [61, 62]. Similarly, champions are often described as devoted individuals who facilitate successful implementation of innovations [63, 64]. Our results showed examples of the influence of champions who received no specific training to exercise that role but who motivated and supported colleagues at the school level. At the municipality level, facilitators, who received no specific training, enhanced sustainment by enforcing program adoption and providing extra support to schools. While champions can support implementation, program sustainability requires formally appointed facilitators and collaboration between school staff [62, 65]. Theoretically speaking, implementing a complex program such as the HSS at scale, i.e., across entire municipalities, generates a degree of interdependence among and between the school staff and the municipality leaders. This interdependence requires effective collaboration and equal distribution of responsibilities to succeed in the long term.

Community actors such as civil society, PHCs and families have been emphasized as important stakeholders potentially influencing the sustainability of school-based health promotion interventions [18]. In our study, participants expressed that the engagement of community actors (e.g., PHC, civil society, sports clubs, and food retailers) was considered necessary for the program to effectively promote healthy habits in the longer perspective. During the planning phase of the IMPROVE study, PHC personnel were involved in the identification of barriers and facilitators. Similarly, promoting network weaving between schools and PHCs was one of the implementation strategies [43]. However, the PHC personnel could not prioritize this activity, therefore, the strategy was not realized. Findings from a study conducted in Hong Kong highlighted that community links and intersectoral collaboration could contribute to staff’s understanding of their role in school-based health promotion programs [66]. Involving the wider community could be important for HSS program implementation, as a sense of isolation was perceived by some of the principals, who expressed that the school alone could not solve the obesity crisis. Strengthening the link between schools and PHC could not only increase the perceived effectiveness of the HSS program but also improve the feeling of support among staff, as highlighted in Theme 5. Furthermore, promoting health across multiple levels and life stages can increase the perceived need for the HSS program among both leaders and staff, fostering their collective commitment and confidence. This can translate into increased readiness for change which in turn could improve the conditions for institutionalization of the program into school and municipality routines.

Feeling supported was crucial for successful implementation and sustainment, as it led to an improved understanding of the staff’s roles in the program, less stress in working with the HSS program in the first year and improved perceived effectiveness. Support for implementation and program sustainment are challenging factors to achieve [18]. In M1, program implementation was paused after two years by the municipality, due to a combination of factors such as changes in the timing of the health counselling from pre-school class to first grade, reorganization in the municipal structure and prioritization of other activities, as reported by the municipality leaders. Interestingly, this occurred after the IMPROVE study had concluded in that municipality. However, we cannot conclude that the shift in priorities was due to the characteristics of the HSS or the implementation experience. Participants in M1 did not report any particularly negative experiences regarding the implementation. A systematic review by Herlitz et al. found that, out of 18 included health promotion programs, none of them were sustained entirely after implementation support by external actors had ended [18]. Moreover, many interventions lack systematic follow-up beyond the initial implementation phase [67] and evidence indicates that teaching staff that works with health promotion interventions need to be supported by their supervisors to avoid being overburdened [37]. Leadership support, can signal progress toward the institutionalization of the HSS program from a theoretical standpoint [30]. This, in turn, can reinforce commitment and accountability, thereby enhancing readiness by showing that the change is valued by the municipality leaders.

The participants in our study thought that if researchers had been present during the meetings with parents in the school, they could have emphasized the importance of working and engaging with the HSS program and could have reduced skepticism among some families. Lack of parent engagement was also identified as a barrier prior to the implementation of the HSS program [53]. From an Organizational Readiness perspective, this could also contribute to the shared sense of confidence to collectively implement and achieve the goals of the HSS program [30]. A lack of prioritization of health promotion by schools, partnerships between the school and external organizations, and supporting policies or guidelines has been reported as barriers of sustainment for nutrition and physical activity programs in primary school in Australia [36]. Importantly, the most frequent facilitator of program sustainment identified by three systematic reviews in terms of support is endorsement to institutionalize and integrate the intervention into the organization or school [18, 68, 69]. This is in line with the municipality leaders’ suggestions to include the HSS in the core program for schools.

More research is needed on effective strategies that increase the feeling of support among school staff as well as the mechanisms and determinants of adoption and sustainment of programs such as the HSS.

### Strengths and limitations

A methodological strength of this study was the well-structured recruitment framework, which ensured transparency and rigor by inviting all municipality leaders and principals involved in the decision-making process and the implementation of the HSS program. This careful mapping and identification of potential participants contributed to the multivocality and supported credibility and dependability [70] in this study. This comprehensive approach in the data collection process is aligned with the Criteria for Excellent Qualitative Research [70]. Similarly, in line with ethical and rigorous qualitative research practices [70], efforts were made to include a group of stakeholders as diverse and broad as possible, despite some participants being unable or willing to participate in the interviews. The concept of information power was applied to determine our sample [49], thereby contributing to the study’s rich rigor [70]. Accordingly, the aim of this study is narrow, the sample specificity was dense, and the Theory of Organizational Readiness for Change [30], was applied which contributed to a higher information power by adding to the depth of the analysis and thereby supporting the generation of relevant and meaningful themes.

The interviews were conducted by one or two researchers either at the participants’ workplace or online. This arrangement gave the participants the role of ‘hosts’ in the interviews, as they could choose the location. Conducting the interviews in person was advantageous, for instance by not having to rely on an internet connection which made communication smoother. However, we observed that participants also appeared comfortable during online interviews, which we believe contributed positively to the quality of the dialogue. Having two researchers present helped in probing and asking follow-up questions that only one researcher might have missed. On the other hand, one-on-one interviews might have made participants feel more comfortable. From our perspective, participants were relaxed and confident during the interviews, which contributed to a high-quality dialog, richness of the data and thereby to a higher information power of the sample.

In terms of positionality, the first author (JAA) is a PhD candidate who joined the research team in the late spring of 2023, when the program implementation had ended in two of the municipalities and was still ongoing in the third municipality. Therefore, JAA had the ability to conduct school visits in M3 as part of the IMPROVE study, providing a chance to become familiar with the work at the schools while maintaining the role of ‘outsider’ since he had not been involved in the planning or implementation phase. This could have potentially contributed to participants being able to speak freely about issues that they faced during the implementation of the program.

Considering the focus of this study, which included municipality leaders and school principals, we were able to capture insights into their priorities and experienced challenges related to the implementation process at scale and toward reaching sustainment. Implementation strategies were designed within the IMPROVE study to address potential barriers and facilitators identified in workshops with school staff prior to the start of the implementation study. Our findings, which are based on the experiences of stakeholders involved in the decision-making process and adoption of the program, can contribute to further refinement of implementation strategies. For instance, participants noted that school nurses reported difficulties to deliver MI-sessions to parents who did not speak Swedish. Efforts to address this barrier in the short term would require additional funding to hire trained personnel capable of performing MI talks [71] in the relevant language or alternatively a form of instant technical translation support. Our study can serve as a guide for practitioners and researchers designing and implementing similar programs in school settings, particularly in contexts with decentralized governance like Sweden, where municipalities oversee school health promotion. However, transferability should be considered in relation to local political priorities, resource availability, and leadership structures. It could have been interesting to analyze the data based on the IMPROVE bundles. However, since municipality leaders operated at a higher level and were not involved in or affected by the bundle-specific strategies, it was not possible to differentiate the analysis between Basic and Enhanced strategies.

Our study was designed to explore the perspectives of school principals and municipality leaders, which did not allow us to capture frontline implementation experiences, i.e. school staff, and family engagement. However, previous qualitative studies on the HSS program have explored these perspectives in depth, specifically among parents [27], children [28], teachers [72] and school nurses [29, 56]. They provide complementary insights into the delivery of the program and the way it was received. Future research could benefit from integrating these views to triangulate findings and further inform implementation strategies.

One limitation of this study is the homogeneous sample, as only female participants participated. All the municipality leaders involved in the decision-making process to implement the HSS program were female. However, nine of the eligible principals were males who were invited to the study; two of them declined participation, and seven did not respond to the invitation even after several reminders. Another limitation was the potential for self-selection bias among participants. Those who agreed to participate may have been more engaged with or supportive of the HSS program, which could influence the perspectives captured in the study. Moreover, one year had elapsed between the implementation in M1 and M2 by the time of the interviews. This could have introduced recall bias among the participants from those two municipalities.

## Conclusion

Success factors for scaling up and sustaining the HSS program include consistent implementation support from leaders in the municipality and school, motivated staff, and the possibility of planning in advance and clearly defining the staff’s roles and responsibilities from the beginning. Barriers to sustainment at scale included difficulties in prioritizing the HSS program over other important tasks at the municipal level and occasionally, challenges in delivering the MI component. Additionally, staff and leadership turnover, particularly when incoming leadership were less committed and motivated toward the implementation of the HSS, posed a challenge to the long-term sustainment of the program. Program integration into existing routines and in the yearly quality assessment might be the missing piece of the puzzle needed to achieve sustained implementation at scale. Our findings are likely to be applicable in settings with a similar decentralized school system as the Swedish.

## Supplementary Information

Additional file 1.

Additional file 2.

Additional file 3.

## Data Availability

The datasets generated and/or analyzed during the current study are not publicly available owing to efforts to protect the confidentiality of participants.

## Abbreviations

HSS: Healthy School Start
BMI: Body mass index
MI: Motivational interviewing
T2D: Type 2 Diabetes
PHC: Primary Health Care
TORC: Theory of Organizational Readiness for Change
COREQ: Consolidated Criteria for Reporting Qualitative Studies Questionnaire
M1: Municipality 1
M2: Municipality 2
M3: Municipality 3
ISAT: Intervention Scalability Assessment Tool

## References

[1] The Lancet Diabetes & Endocrinology. Childhood obesity: a growing pandemic. Lancet Diabetes Endocrinol. 2022;10:1. 10.1016/S2213-8587(21)00314-4. 34863372 10.1016/S2213-8587(21)00314-4 PMC9765420

[2] Chong B, Jayabaskaran J, Kong G, Chan YH, Chin YH, Goh R, et al. Trends and predictions of malnutrition and obesity in 204 countries and territories: an analysis of the global burden of disease study 2019. eClinicalMedicine. 2023;57. 10.1016/j.eclinm.2023.101850. 10.1016/j.eclinm.2023.101850 PMC9971264 36864983

[3] Zhang X, Liu J, Ni Y, Yi C, Fang Y, Ning Q, et al. Global prevalence of overweight and obesity in children and adolescents: a systematic review and Meta-Analysis. JAMA Pediatr. 2024;178:800–13. 10.1001/jamapediatrics.2024.1576. 38856986 10.1001/jamapediatrics.2024.1576 PMC11165417

[4] World Health Organization. Obesity and overweight. https://www.who.int/news-room/fact-sheets/detail/obesity-and-overweight. Accessed 3 Dec 2024.

[5] The Public Health Agency of Sweden. Statistik om övervikt och fetma hos barn 6–10 år. 2024. https://www.folkhalsomyndigheten.se/livsvillkor-levnadsvanor/mat-fysisk-aktivitet-overvikt-och-fetma/overvikt-och-fetma/statistik-om-overvikt-och-fetma/overvikt-och-fetma-hos-barn-6-10-ar/. Accessed 3 Dec 2024.

[6] Juhola J, Magnussen CG, Viikari JSA, Kähönen M, Hutri-Kähönen N, Jula A, et al. Tracking of serum lipid levels, blood pressure, and body mass index from childhood to adulthood: the cardiovascular risk in young Finns study. J Pediatr. 2011;159:584–90. 10.1016/j.jpeds.2011.03.021. 21514597 10.1016/j.jpeds.2011.03.021

[7] Lee K, van Nassau F, Grunseit A, Conte K, Milat A, Wolfenden L, et al. Scaling up population health interventions from decision to sustainability – a window of opportunity? A qualitative view from policy-makers. Health Res Policy Sys. 2020;18:118. 10.1186/s12961-020-00636-3. 10.1186/s12961-020-00636-3 PMC7547476 33036633

[8] Okunogbe A, Nugent R, Spencer G, Ralston J, Wilding J. Economic impacts of overweight and obesity: current and future estimates for eight countries. BMJ Global Health. 2021;6:e006351. 10.1136/bmjgh-2021-006351. 34737167 10.1136/bmjgh-2021-006351 PMC8487190

[9] Brown T, Moore TH, Hooper L, Gao Y, Zayegh A, Ijaz S et al. Interventions for preventing obesity in children. Cochrane Database of Systematic Reviews. 2019;7:CD001871.10.1002/14651858.CD001871.pub4. 10.1002/14651858.CD001871.pub4 PMC6646867 31332776

[10] Gori D, Guaraldi F, Cinocca S, Moser G, Rucci P, Fantini MP. Effectiveness of educational and lifestyle interventions to prevent paediatric obesity: systematic review and meta-analyses of randomized and non-randomized controlled trials. Obes Sci Pract. 2017;3:235–48. 10.1002/osp4.111. 29071100 10.1002/osp4.111 PMC5649699

[11] Drouka A, Brikou D, Causeret C, Al Ali Al Malla N, Sibalo S, Ávila C, et al. Effectiveness of School-Based interventions in Europe for promoting healthy lifestyle behaviors in children. Children. 2023;10:1676. 10.3390/children10101676. 37892339 10.3390/children10101676 PMC10605522

[12] Jacob CM, Hardy-Johnson PL, Inskip HM, Morris T, Parsons CM, Barrett M, et al. A systematic review and meta-analysis of school-based interventions with health education to reduce body mass index in adolescents aged 10 to 19 years. Int J Behav Nutr Phys Act. 2021;18:1. 10.1186/s12966-020-01065-9. 33397403 10.1186/s12966-020-01065-9 PMC7784329

[13] Liu Z, Xu H-M, Wen L-M, Peng Y-Z, Lin L-Z, Zhou S, et al. A systematic review and meta-analysis of the overall effects of school-based obesity prevention interventions and effect differences by intervention components. Int J Behav Nutr Phys Act. 2019;16:95. 10.1186/s12966-019-0848-8. 31665040 10.1186/s12966-019-0848-8 PMC6819386

[14] Aleid AM, Sabi NM, Alharbi GS, Alharthi AA, Alshuqayfi SM, Alnefiae NS, et al. The impact of parental involvement in the prevention and management of obesity in children: a systematic review and meta-analysis of randomized controlled trials. Children. 2024;11:739. 10.3390/children11060739. 38929318 10.3390/children11060739 PMC11201836

[15] Summerbell CD, Moore HJ, Vögele C, Kreichauf S, Wildgruber A, Manios Y, et al. Evidence-based recommendations for the development of obesity prevention programs targeted at preschool children. Obes Rev. 2012;13:129–32. 10.1111/j.1467-789X.2011.00940.x. 22309071 10.1111/j.1467-789X.2011.00940.x

[16] Koorts H, Rutter H. A systems approach to scale-up for population health improvement. Health Res Policy Sys. 2021;19:27. 10.1186/s12961-021-00679-0. 10.1186/s12961-021-00679-0 PMC7919988 33648525

[17] McCrabb S, Lane C, Hall A, Milat A, Bauman A, Sutherland R, et al. Scaling-up evidence-based obesity interventions: a systematic review assessing intervention adaptations and effectiveness and quantifying the scale-up penalty. Obes Rev. 2019;20:964–82. 10.1111/obr.12845. 30868745 10.1111/obr.12845

[18] Herlitz L, MacIntyre H, Osborn T, Bonell C. The sustainability of public health interventions in schools: a systematic review. Implement Sci. 2020;15:4. 10.1186/s13012-019-0961-8. 31906983 10.1186/s13012-019-0961-8 PMC6945701

[19] Darlington EJ, Violon N, Jourdan D. Implementation of health promotion programmes in schools: an approach to understand the influence of contextual factors on the process? BMC Public Health. 2018;18:163. 10.1186/s12889-017-5011-3. 29357922 10.1186/s12889-017-5011-3 PMC5776776

[20] Wolfenden L, Foy R, Presseau J, Grimshaw JM, Ivers NM, Powell BJ, et al. Designing and undertaking randomised implementation trials: guide for researchers. BMJ. 2021;372:m3721. 10.1136/bmj.m3721. 33461967 10.1136/bmj.m3721 PMC7812444

[21] Elinder LS, Patterson E, Nyberg G, Norman Å. A healthy school start plus for prevention of childhood overweight and obesity in disadvantaged areas through parental support in the school setting - study protocol for a parallel group cluster randomised trial. BMC Public Health. 2018;18:459. 10.1186/s12889-018-5354-4. 29625599 10.1186/s12889-018-5354-4 PMC5889522

[22] Norman Å, Malek ME, Nyberg G, Patterson E, Elinder LS. Effects of universal school-based parental support for children’s healthy diet and physical activity—the healthy school start plus cluster–randomised controlled trial. Prev Sci. 2024;25:963–77. 10.1007/s11121-024-01697-4. 38987407 10.1007/s11121-024-01697-4 PMC11390772

[23] Nyberg G, Norman Å, Sundblom E, Zeebari Z, Elinder LS. Effectiveness of a universal parental support programme to promote health behaviours and prevent overweight and obesity in 6-year-old children in disadvantaged areas, the healthy school start study II, a cluster-randomised controlled trial. Int J Behav Nutr Phys Activity. 2016;13:4. 10.1186/s12966-016-0327-4. 10.1186/s12966-016-0327-4 PMC4721005 26795378

[24] Nyberg G, Sundblom E, Norman Å, Bohman B, Hagberg J, Elinder LS. Effectiveness of a universal parental support programme to promote healthy dietary habits and physical activity and to prevent overweight and obesity in 6-year-old children: the healthy school start study, a cluster-randomised controlled trial. PLoS ONE. 2015;10:e0116876. 10.1371/journal.pone.0116876. 25680096 10.1371/journal.pone.0116876 PMC4332680

[25] Patterson E, Nyberg G, Norman Å, Schäfer Elinder L. Universal healthy school start intervention reduced the body mass index of young children with obesity. Acta Paediatrica. n/a n/a. 10.1111/apa.17164. 10.1111/apa.17164 38381539

[26] Sweden’s Municipalities and Regions. Kommuner och regioner. https://skr.se/skr/tjanster/kommunerochregioner.431.html. Accessed 24 Mar 2025.

[27] Malek ME, Andermo S, Nyberg G, Elinder LS, Patterson E, Norman Å. Parents’ experiences of participating in the healthy school start plus programme – a qualitative study. BMC Public Health. 2023;23:646. 10.1186/s12889-023-15552-8. 37016372 10.1186/s12889-023-15552-8 PMC10074815

[28] Malek ME, Nyberg G, Elinder LS, Patterson E, Norman Å. Children’s experiences of participating in a school-based health promotion parental support programme – a qualitative study. BMC Pediatr. 2021;21:228. 10.1186/s12887-021-02694-0. 33975569 10.1186/s12887-021-02694-0 PMC8111964

[29] Moberg M, Lindqvist H, Andermo S, Norman Å. Sometimes it felt great, and sometimes it just went pear-shaped: experiences and perceptions of school nurses’ motivational interviewing competence: a convergent mixed-methods study. Clin Pract. 2022;12:333–49. 10.3390/clinpract12030039. 35645316 10.3390/clinpract12030039 PMC9149896

[30] Weiner BJ. A theory of organizational readiness for change. Implement Sci. 2009;4:67. 10.1186/1748-5908-4-67. 19840381 10.1186/1748-5908-4-67 PMC2770024

[31] Walker TJ, Brandt HM, Wandersman A, Scaccia J, Lamont A, Workman L, et al. Development of a comprehensive measure of organizational readiness (motivation × capacity) for implementation: a study protocol. Implement Sci Commun. 2020;1:103. 10.1186/s43058-020-00088-4. 33292840 10.1186/s43058-020-00088-4 PMC7656510

[32] Weiner BJ, Amick H, Lee S-YD, Review. Conceptualization and measurement of organizational readiness for change: a review of the literature in health services research and other fields. Med Care Res Rev. 2008;65:379–436. 10.1177/1077558708317802. 18511812 10.1177/1077558708317802

[33] Scaccia JP, Cook BS, Lamont A, Wandersman A, Castellow J, Katz J, et al. A practical implementation science heuristic for organizational readiness: R = Mc2. J Community Psychol. 2015;43:484–501. 10.1002/jcop.21698. 26668443 10.1002/jcop.21698 PMC4676714

[34] Durlak JA, DuPre EP. Implementation matters: A review of research on the influence of implementation on program outcomes and the factors affecting implementation. Am J Community Psychol. 2008;41:327–50. 10.1007/s10464-008-9165-0. 18322790 10.1007/s10464-008-9165-0

[35] Shelton RC, Cooper BR, Stirman SW The sustainability of evidence-based interventions and practices in public health and health care. Ann Rev Pub Health. 2018;39:55–76. 10.1146/annurev-publhealth-040617-014731. 10.1146/annurev-publhealth-040617-014731 29328872

[36] Riley-Gibson E, Hall A, Shoesmith A, Shelton RC, Lecathelinais C, Hodder RK, et al. Identifying key determinants influencing the sustainment of physical activity and nutrition programs in Australian primary schools. Int J Behav Nutr Phys Activity. 2025;22:116. 10.1186/s12966-025-01808-6. 10.1186/s12966-025-01808-6 PMC12399001 40885977

[37] Day RE, Sahota P, Christian MS. Effective implementation of primary school-based healthy lifestyle programmes: a qualitative study of views of school staff. BMC Public Health. 2019;19:1239. 10.1186/s12889-019-7550-2. 31500603 10.1186/s12889-019-7550-2 PMC6734437

[38] Adamowitsch M, Gugglberger L, Dür W. Implementation practices in school health promotion: findings from an Austrian multiple-case study. Health Promot Int. 2017;32:218–30. 10.1093/heapro/dau018. 24682544 10.1093/heapro/dau018

[39] Braun V, Clarke V. One size fits all? What counts as quality practice in (reflexive) thematic analysis? Qualitative. Res Psychol. 2021;18:328–52. 10.1080/14780887.2020.1769238.

[40] Braun V. Using thematic analysis in psychology. Qualitative Res Psychol. 2006;3:77–101. 10.1191/1478088706qp063oa.

[41] Braun V, Clarke V. Toward good practice in thematic analysis: avoiding common problems and be(com)ing a knowing researcher. Int J Transgender Health. 2023;24:1–6. 10.1080/26895269.2022.2129597. 10.1080/26895269.2022.2129597 PMC9879167 36713144

[42] Tong A, Sainsbury P, Craig J. Consolidated criteria for reporting qualitative research (COREQ): a 32-item checklist for interviews and focus groups. Int J Qual Health Care. 2007;19:349–57. 10.1093/intqhc/mzm042. 17872937 10.1093/intqhc/mzm042

[43] Elinder LS, Wiklund CA, Norman Å, Stattin NS, Andermo S, Patterson E, et al. IMplementation and evaluation of the school-based family support program a healthy school start to promote child health and prevent overweight and obesity (IMPROVE) – study protocol for a cluster-randomized trial. BMC Public Health. 2021;21:1630. 10.1186/s12889-021-11663-2. 34488691 10.1186/s12889-021-11663-2 PMC8419825

[44] Statistics Sweden. Folkmängden per månad efter region och månad. https://www.statistikdatabasen.scb.se/pxweb/sv/ssd/START__BE__BE0101__BE0101A/BefolkManad/table/tableViewLayout1/?loadedQueryId=139767&timeType=top&timeValue=1. Accessed 3 Nov 2024.

[45] Swedish Public Employment Agency. Statistik arbetslöshet och arbetssökande. Arbetsförmedlingen. https://arbetsformedlingen.se/statistik. Accessed 3 Dec 2024.

[46] Statistics Sweden. Befolkning efter region, ålder, utbildningsnivå och år. https://www.statistikdatabasen.scb.se/pxweb/sv/ssd/START__UF__UF0506__UF0506B/Utbildning/table/tableViewLayout1/?loadedQueryId=155433&timeType=item. Accessed 3 Dec 2024.

[47] Child Health Care Unit, Region Stockholm [Barnhälsovårdsenheten i Region Stockholm]. Annual report of child health care in Stockholm County, 2022 [Årsrapport Barnhälsovård i Stockholms län 2022]. Stockholm: Region Stockholm. 2023. Available from: https://kunskapsstodforvardgivare.se/download/18.3bc843dd18851928cf725bb8/1686724139915/2022_%C3%85rsrapport.pdf.

[48] Patton MQ. Qualitative research & evaluation methods: integrating theory and practice. 4th edition. SAGE; 2014. p. 266-268, Qualitative designs and data collection – purposeful sampling strategies.

[49] Malterud K, Siersma VD, Guassora AD. Sample size in qualitative interview studies: guided by information power. Qual Health Res. 2016;26:1753–60. 10.1177/1049732315617444. 26613970 10.1177/1049732315617444

[50] Milat A, Lee K, Conte K, Grunseit A, Wolfenden L, van Nassau F, et al. Intervention scalability assessment tool: A decision support tool for health policy makers and implementers. Health Res Policy Sys. 2020;18:1. 10.1186/s12961-019-0494-2. 10.1186/s12961-019-0494-2 PMC6942323 31900230

[51] Braun V, Clarke V. Successful qualitative research : a practical guide for Beginners. Los Angeles: SAGE; 2013.

[52] Nadeem E, Ringle VA. De-adoption of an Evidence-Based trauma intervention in schools: a retrospective report from an urban school district. School Mental Health. 2016;8:132–43. 10.1007/s12310-016-9179-y. 28775793 10.1007/s12310-016-9179-y PMC5538780

[53] Bergström H, Sundblom E, Elinder LS, Norman Å, Nyberg G. Managing implementation of a parental support programme for obesity prevention in the school context: the importance of creating commitment in an overburdened work Situation, a qualitative study. J Prim Prevent. 2020;41:191–209. 10.1007/s10935-020-00584-2. 10.1007/s10935-020-00584-2 PMC7230040 32157622

[54] Jakobsson M, Moberg M. School nurses’ perceived capability, opportunity, and motivation to provide health promotion: a convergent mixed-methods study. Int J Nurs Stud Adv. 2025;9:100396. 10.1016/j.ijnsa.2025.100396. 40822249 10.1016/j.ijnsa.2025.100396 PMC12356015

[55] Goh TL, Hannon JC, Webster CA, Podlog L. Classroom teachers’ experiences implementing a movement integration program: barriers, facilitators, and continuance. Teach Teacher Educ. 2017;66:88–95. 10.1016/j.tate.2017.04.003.

[56] Moberg M, Norman Å, Schäfer Elinder L, Andermo S. School nurses’ perceptions and experiences of delivering a universal health-promotion program targeting both children and parents in the Swedish primary school context. BMC Nurs. 2025;24:1158. 10.1186/s12912-025-03806-2. 40898241 10.1186/s12912-025-03806-2 PMC12406544

[57] Samad N, Bearne L, Noor FM, Akter F, Parmar D. School-based healthy eating interventions for adolescents aged 10–19 years: an umbrella review. Int J Behav Nutr Phys Act. 2024;21:117. 10.1186/s12966-024-01668-6. 39402562 10.1186/s12966-024-01668-6 PMC11472496

[58] Swedish National Agency for Education. Ansvar för systematiskt kvalitetsarbete (Responsibility for the systematic quality assurance). https://www.skolverket.se/regler-och-ansvar/ansvar-i-skolfragor/ansvar-for-systematiskt-kvalitetsarbete. Accessed 19 Dec 2024.

[59] Tjomsland H, Larsen TMB, Viig NG, Wold B. A fourteen year Follow-up study of health promoting schools in Norway: principals` perceptions of conditions influencing sustainability. Open Educ J. 2009. 10.2174/1874920800902010054.

[60] Dogherty EJ, Harrison MB, Graham ID. Facilitation as a role and process in achieving evidence-based practice in nursing: a focused review of concept and meaning. Worldviews Evidence-Based Nurs. 2010;7:76–89. 10.1111/j.1741-6787.2010.00186.x. 10.1111/j.1741-6787.2010.00186.x 20180826

[61] Yakovchenko V, Merante M, Chinman MJ, Neely B, Lamorte C, Gibson S, et al. The good enough facilitator: elucidating the role of working alliance in the mechanism of facilitation. Implement Sci Commun. 2025;6:22. 10.1186/s43058-025-00705-0. 40001234 10.1186/s43058-025-00705-0 PMC11863522

[62] Olmos-Ochoa TT, Ganz DA, Barnard JM, Penney L, Finley EP, Hamilton AB, et al. Sustaining implementation facilitation: a model for facilitator resilience. Implement Sci Commun. 2021;2:65. 10.1186/s43058-021-00171-4. 34154670 10.1186/s43058-021-00171-4 PMC8218441

[63] Santos WJ, Graham ID, Lalonde M, Demery Varin M, Squires JE. The effectiveness of champions in implementing innovations in health care: a systematic review. Implement Sci Commun. 2022;3:80. 10.1186/s43058-022-00315-0. 35869516 10.1186/s43058-022-00315-0 PMC9308185

[64] Astorino Nicola J, Nataliansyah MM, Lopez-Olivo MA, Adegboyega A, Hirko KA, Chichester L-AR, et al. Champions to enhance implementation of clinical and community-based interventions in cancer: a scoping review. Implement Sci Commun. 2024;5:119. 10.1186/s43058-024-00662-0. 39439009 10.1186/s43058-024-00662-0 PMC11494796

[65] Harvey G, Kitson A. PARIHS revisited: from heuristic to integrated framework for the successful implementation of knowledge into practice. Implement Sci. 2016;11:33. 10.1186/s13012-016-0398-2. 27013464 10.1186/s13012-016-0398-2 PMC4807546

[66] Lee A, Lo ASC, Keung MW, Kwong CMA, Wong KK. Effective health promoting school for better health of children and adolescents: indicators for success. BMC Public Health. 2019;19:1088. 10.1186/s12889-019-7425-6. 31409312 10.1186/s12889-019-7425-6 PMC6691553

[67] Wiltsey Stirman S, Kimberly J, Cook N, Calloway A, Castro F, Charns M. The sustainability of new programs and innovations: a review of the empirical literature and recommendations for future research. Implement Sci. 2012;7:1–19. 10.1186/1748-5908-7-17. 10.1186/1748-5908-7-17 PMC3317864 22417162

[68] Shoesmith A, Hall A, Wolfenden L, Shelton RC, Powell BJ, Brown H, et al. Barriers and facilitators influencing the sustainment of health behaviour interventions in schools and childcare services: a systematic review. Implement Sci. 2021;16:62. 10.1186/s13012-021-01134-y. 34118955 10.1186/s13012-021-01134-y PMC8199827

[69] Cassar S, Salmon J, Timperio A, Naylor P-J, van Nassau F, Contardo Ayala AM, et al. Adoption, implementation and sustainability of school-based physical activity and sedentary behaviour interventions in real-world settings: a systematic review. Int J Behav Nutr Phys Act. 2019;16:120. 10.1186/s12966-019-0876-4. 31791341 10.1186/s12966-019-0876-4 PMC6889569

[70] Tracy SJ. Qualitative quality: eight big-tent criteria for excellent qualitative research. Qualitative Inq. 2010;16:837–51. 10.1177/1077800410383121.

[71] Powell BJ, Waltz TJ, Chinman MJ, Damschroder LJ, Smith JL, Matthieu MM, et al. A refined compilation of implementation strategies: results from the expert recommendations for implementing change (ERIC) project. Implement Sci. 2015;10:21. 10.1186/s13012-015-0209-1. 25889199 10.1186/s13012-015-0209-1 PMC4328074

[72] Bergström H, Haggård U, Norman Å, Sundblom E, Schäfer Elinder L, Nyberg G. Factors influencing the implementation of a school-based parental support programme to promote health-related behaviours—interviews with teachers and parents. BMC Public Health. 2015;15:541. 10.1186/s12889-015-1896-x. 26051650 10.1186/s12889-015-1896-x PMC4459678

