# Supplementary material for: “The missing piece in the puzzle” - Success factors and barriers for scale-up and sustainment of the Healthy School Start program
Source: Arch Public Health. 2026 Jan 9;84:20. doi: 10.1186/s13690-026-01835-0 (PMC12849295; doi:10.1186/s13690-026-01835-0)
Supplement: Supplementary file 1 — Additional file 1. [file 13690_2026_1835_MOESM1_ESM.docx]

APPENDIX 1 - COREQ checklist

The Consolidated Criteria for Reporting Qualitative Studies (COREQ): 32-item checklist

| No. Item | Guide questions/description | Notes |
| --- | --- | --- |
| Domain 1: Research team and reﬂexivity | | |
| The research team  JAA, KSA, LSE, SA | | |
|  | | |
| Personal Characteristics | | |
| 1. Inter  viewer/facilitator | Which author/s conducted the interview or focus group? | JAA conducted all semi-structured interviews. The co-authors were present in some of the interviews: KSA in 3, LSE in 3 and SA in 2 interviews. |
| 2. Credentials | What were the researcher’s  credentials? E.g. PhD, MD | JAA: PhD student, KSA: PhD, LSE: PhD, SA: PhD |
| 3. Occupation | What was their occupation at the time of the study? | JAA: PhD student, KSA and SA Associate Professors; and LSE Professor. |
| 4. Gender | Was the researcher male or female? | The interviewer (JAA) is male. KSA, LSE and SA are female. |
| 5. Experience and training | What experience or training did the researcher(s) have? | JAA has little practical experience with qualitative methods, he took qualitative courses prior to the interviews.  KSA, LSE and SA have extensive experience with qualitative methods including conducting interviews and have authored qualitative research papers. |
| Relationship with participants | | |
| 6. Relationship established | Was a relationship established prior to study commencement? | LSE and KSA had had contact with the interview participants previously related to the implementation of the HSS program. JAA had been present at a few meetings regarding the implementation of the program. SA had not had previous contact with the participants. |
| 7. Participant knowledge of the interviewer | What did the participants know about the researcher? e.g. personal goals, reasons for doing the research | Participants were aware that the research project aimed at evaluating the implementation of the HSS at their municipality/school. The research team explained the purpose of the interviews and of this study. |
| 8. Interviewer characteristics | What characteristics were reported about the inter viewer/facilitator?  e.g. Bias, assumptions, reasons and interests in the research topic | The research team had an interest in understanding the factors that influenced the implementation of the HSS. |
| Domain 2: Study design | | |
| Theoretical framework | | |
| 9. Methodological orientation and Theory | What methodological orientation was stated to underpin the study?  e.g. grounded theory, discourse analysis, ethnography, phenomenology, content analysis | The ontology for this study has manifestations of constructivism, our main focus was on understanding how participants perceived program implementation. Data was analyzed inductively applying reflexive thematic analysis as described by Braun and Clark. |

| 10. Sampling | How were participants selected? e.g. purposive, convenience, consecutive, snowball | We applied purposeful sample. We deliberately selected participants that had good knowledge and previous experience of implementing the HSS and could provide substantial information based on their experience. Heads of unit and other employees at the municipality administration, as well as school principals and adjuncts that were involved in the implementation of the HSS were invited for interviews. |
| --- | --- | --- |
| 11. Method of approach | How were participants approached?  e.g. face-to-face, telephone, mail, email | Participants from schools in the two municipalities that had already worked with the HSS were contacted via email the first time, then two rounds of calls and a final email reminder.  Participants from schools in the municipality that was implementing the HSS program at the time of recruitment, were contacted during meetings that were part of the implementation study.  All municipality staff were reached via email. |
| 12. Sample size | How many participants were in the study? | 8 municipality leaders and 8 school principals or adjunct principals. |
| 13. Non-participation | How many people refused to participate or dropped out? Reasons? | The total number of eligible municipality leaders involved in the implementation was 10 of which 8 were interviewed and 2 refused to participate due to a lack of time. There were 43 eligible schools who had implemented the HSS, in most cases, there was a principal and an adjunct principal working at the school. In total, 43 principals and 31 vice principals were invited, they could decide which of them would participate depending on who had been involved in the HSS. In total 13 were no longer working at the school, 17 were not involved with the HSS, 7 refused to participate, 29 did not reply to the invitation after three reminders |
| Setting | | |
| 14. Setting of data collection | Where was the data collected? e.g. home, clinic, workplace | Two out of eight interviews with municipality leaders were conducted online and the other six were conducted at the participants’ office. Three out of eight interviews with school principals were conducted online and the other five were done at the schools. Remote interviews were done this way upon the participants’ request |
| 15. Presence of non- participants | Was anyone else present besides the participants and researchers? | No one else was present during the data collection besides participants and researchers. |
| 16. Description of sample | What are the important characteristics of the sample? e.g. demographic data, date | All participants were female, aged between 36 and 64 years, held university degrees and had been working in their current position for 5 years on average |
| Data collection | | |
| 17. Interview guide | Were questions, prompts, guides provided by the authors? Was it pilot tested? | An interview guide was developed to understand success factors for implementation, scale up and sustainment based on the Intervention Scalability Assessment Tool (Milat et al 2020). The interview guide was tested among the researchers and colleagues. |
| 18. Repeat interviews | Were repeat inter views carried out? If yes, how many? | There were no repeat interviews with the same participants. |
| 19. Audio/visual recording | Did the research use audio or visual recording to collect the data? | All interviews were audio recorded with permission of participants. |
| 20. Field notes | Were ﬁeld notes made during and/or after the interview or focus group? | Researchers made field notes during the interviews to capture non-verbal information that took place during the interviews. The field notes were taken into consideration in the data analysis |

| 21. Duration | What was the duration of the interviews or focus group? | The interviews had an average duration of 42 minutes |
| --- | --- | --- |
| 22. Data saturation | Was data saturation discussed? | The concept of information power (Malterud et al 2015) was applied to determine the sample size. |
| 23. Transcripts returned | Were transcripts returned to participants for comment and/or correction? | Transcripts were not returned to participants for comment and/or correction. |
| Domain 3: Analysis and findings | | |
| Data analysis | | |
| 24. Number of data coders | How many data coders coded the data? | The coding was initially done by one researcher (JAA) and discussed in iterations with all the co-authors. The codes were discussed reflectively and adjusted if necessary. |
| 25. Description of the coding tree | Did authors provide a description of the coding tree? | The codes were grouped, condensed explanations giving meaning to the codes were written, these were grouped, and potential sub-themes were generated |
| 26. Derivation of themes | Were themes identified in advance or derived from the data? | Themes were generated from the codes inductively |
| 27. Software | What software, if applicable, was used to manage the data? | Microsoft Word and Excel |
| 28. Participant checking | Did participants provide feedback on the findings? | There was no member check performed |
| Reporting | | |
| 29. Quotations presented | Were participant quotations presented to illustrate the themes/ findings? Was each quotation identified? E.g. participant number | Key findings of this study are supported with quotes and identified by participant number. No names or personal information on the participants is disclosed. |
| 30. Data and findings consistent | Was there consistency between the data presented and the findings? | All findings are based on the interview data and the reflexive thematic analysis performed. The themes and sub-themes generated are supported by quotes to illustrate the findings. |
| 31. Clarity of major themes | Were major themes clearly presented in the findings? | Major themes were derived from the data and are clearly defined by a paragraph title. |
| 32. Clarity of minor themes | Is there a description of diverse cases or discussion of minor themes? | Sub-themes were generated with components that constituted the main elements of the themes and gave them depth. |
