# Supplementary material for: “The missing piece in the puzzle” - Success factors and barriers for scale-up and sustainment of the Healthy School Start program
Source: Arch Public Health. 2026 Jan 9;84:20. doi: 10.1186/s13690-026-01835-0 (PMC12849295; doi:10.1186/s13690-026-01835-0)
Supplement: Supplementary file 2 — Additional file 2. [file 13690_2026_1835_MOESM2_ESM.docx]

**Intervjuguide för ledande personer i kommunen och skolan**

- Berätta om syftet med intervjun, ämnet och ge en chans att reflektera och förstå hur En Frisk Skolstart (EFS) och IMPROVE studien uppfattas/har upplevts.
- Gå igenom informationsbrev och berätta om EFS och IMPROVE.

**Introduktionsfrågor**

A2: Programmet

- Vad är din roll i din organisation? Vad är din roll i EFS?
- Hur länge har du haft den rollen?
  - Organogram (kommunanställda)
- **Vad tycker du om EFS i stort**?
- Hur kom det sig att ni (i din skola/din kommun) övervägde att gå med/gick med i IMPROVE studien?
- Hur upplevde du att EFS fungerade i stort?
  - Varför? Varför inte?

A1: Hälsoproblemet

- Vad är ditt intryck av barns matvanor, fysisk aktivitet, övervikt och obesitas i din skola/kommun?

A3: Politisk kontext

- Finns det hinder för genomförandet av EFS i din skola/kommun?
  - Hur kan dessa hinder undanröjas?
- Hur anser du att EFS ligger i linje med kommunens politik och intentioner vad gäller hälsofrämjande insatser för barn? Utveckla gärna
- (Kommunal tjänsteman/kvinna) Vilka aktörer från kommunen tycker du bör vara inblandad i implementeringen av EFS? Varför?
- (Endast till kommuner som har redan implementerat EFS) Hur tycker du att covid-19 påverkade implementeringen av EFS?

A4: Evidens för effektivitet

- Utifrån den kunskap du har om programmet och dess effekter, tänker du att det finns tillräckligt stöd (bevis) att programmet kan uppfylla sitt syfte? På vilket sätt?

A5: Kostnad-benefit

- Vilka resurser anser du behövs för att implementera EFS långsiktigt i din kommun? (if any)

**Del B**

B1: Följsamhet och anpassningar

- **Är det något som saknas i EFS-komponenterna?**
- **Vad behövs för att kommunen kan implementera EFS i alla skolor (alltså i större skala)? (kommunal tjänsteman)**
- Behövs anpassningar i programmet för att det ska implementeras långsiktigt? Om ja, vilka?
- Hur tycker du att skolan/kommunen bör följa upp effekten och genomförandet av EFS programmet på längre sikt?

B2: Räckvid och acceptans

- Vad har du för upplevelse av skolpersonalens erfarenheter av att arbete med programmet?
- Hur upplever du att elever och föräldrar tar emot programmet?

B3: Miljö och personal

- (Kommunal tjänsteman/kvinna) Kan du beskriva din relation till ledningen i skolorna i din kommun?

Eller

(Skolpersonal) Kan du beskriva din relation till kommunala ledningen?

- Anser du att det ingår i skolans uppdrag att arbeta med ett hälsofrämjande program som EFS?
- **Hur fungerar samarbetet kring programmet med skolorna?**

B4: Infrastruktur för implementeringen

- Hur är elevhälsan organiserat i din kommun?
- **Hur ser beslutsgången (top down eller bottom up?) ut i din kommun när man överväger att implementera ett nytt program som EFS?**
- Skulle det behövas mer stöd vid uppstart av programmet?
- Saknas något i din skola/kommun för optimal implementering av EFS t.ex. personal, pengar, externt stöd, utrymme?

B5: Hållbarhet

- **Vad skulle kommunen behöva göra för att implementera EFS i alla skolor långsiktigt?**
- Hur kan skolan eller annan aktör upprätthålla kunskapen i familjerna efter genomfört program?

Andra kommenterar?

**Slutligen**

* Ålder och utbildning
