# Supplementary material for: “The missing piece in the puzzle” - Success factors and barriers for scale-up and sustainment of the Healthy School Start program
Source: Arch Public Health. 2026 Jan 9;84:20. doi: 10.1186/s13690-026-01835-0 (PMC12849295; doi:10.1186/s13690-026-01835-0)
Supplement: Supplementary file 3 — Additional file 3. [file 13690_2026_1835_MOESM3_ESM.docx]

**Interview guide for school principals and municipality leaders**

- Explain the purpose of the interview, the topic, and give the participant a chance to reflect and understand how A Healthy School Start (HSS) and the IMPROVE study are perceived/experienced.
- Go through the information letter and explain EFS and IMPROVE.

**Introductory questions**

A2: The program

- What is your role in your organization? What is your role in HSS?
- How long have you had that role?
  - Show organogram
- **What do you think about EFS overall?**
- How did it come about that you (in your school/your municipality) considered joining the IMPROVE study?
- How did you perceive that HSS functioned overall?
  - Why? Why not?

**Part A**

A1: The Health Problem

- What is your impression of children's eating habits, physical activity and the situation with overweight, and obesity in your school/municipality?

A2: The program

- **What do you think about EFS overall?**
- Is there anything missing?
- Implementation Strategy 1: Explain and ask her/him to reflect on the strategy and how it has worked in the municipality.

A3: Political context

- Are there any barriers to implementation?
  - How can these barriers be removed?
- How do you consider that HSS aligns with the municipality’s policies and intentions regarding health promotion initiatives for children?
- (To Municipality leaders) Which actors from the municipality do you think should be involved in the implementation of a HSS? Why?
- *(Only for municipalities that have already implemented HSS)* How do you think COVID-19 affected the implementation of a HSS?

A4: Evidence of effectiveness

- Based on the knowledge you have about the program and its effects, do you think there is sufficient support (evidence) that the program can fulfill its purpose? In what way?

A5: Intervention costs and benefits

- What resources do you think are needed to implement EFS long-term in your municipality? (if any)

**Part B**

B1: Fidelity and adaptation

- Is there anything missing in the HSS components? What?
- What is needed for the municipality to implement HSS in all schools (i.e., on a larger scale)? *(municipality leader)*
- Are adaptations needed in the program for it to be implemented long-term? If yes, which ones?
- How do you think the school/municipality should follow up on the effects and implementation of the HSS program in the long term?

B2: Reach and acceptability

- What is your experience of the school staff’s experiences working with the program?
- How do you perceive that students and parents perceive the program?

B3: Delivery setting and workforce

- (Municipality leader) Can you describe your relationship with the school leadership in your municipality?

or

- (School staff) Can you describe your relationship with the municipal leadership?
- Do you believe it is part of the school’s mission to work with a health-promoting program like the HSS? In what way?
- **How does the collaboration around the program between the municipality and the schools?**

B4: Implementation infrastructure

- How are the school health services organized in your municipality?
- **What does the decision-making process look like (top-down or bottom-up) in your municipality when considering implementing a new program like the HSS?**
- Would more support be needed at the beginning of the program implementation?
- Is anything missing in your school/municipality for optimal implementation of the HSS, e.g., staff, funding, external support, space?

B5: Sustainability of the intervention

- What would the municipality need to do to implement HSS in all schools in the long term?
- How can the school or another actor maintain the knowledge gained through the program among families after the program has been completed?

Other comments?

*Age and education
